# A new approach to ‘on-demand’ treatment of lifelong premature ejaculation by treatment with a combination of a 5-HT_1A_ receptor antagonist and SSRI in rats

**DOI:** 10.3389/fnins.2023.1224959

**Published:** 2023-09-13

**Authors:** Jocelien D. A. Olivier, Josien A. Janssen, Diana C. Esquivel-Franco, Stephen de Prêtre, Berend Olivier

**Affiliations:** ^1^Neurobiology, Groningen Institute for Evolutionary Life Sciences, University of Groningen, Groningen, Netherlands; ^2^Atlas Pharmaceuticals BV, Bruges, Belgium; ^3^Psychopharmacology, Utrecht Institute for Pharmaceutical Sciences, Utrecht University, Utrecht, Netherlands; ^4^Department of Psychiatry, Yale University School of Medicine, New Haven, CT, United States

**Keywords:** premature ejaculation, on demand treatment, SSRI, Atlas987, 5-HT_1A_-receptor antagonist, male, rat, sexual behavior

## Abstract

Lifelong premature ejaculation (PE) in men lacks an adequate on-demand pharmacological treatment. Although selective serotonin reuptake inhibitors (SSRIs) are used for PE they only work after chronic treatment, or if used on-demand, less adequately than chronic SSRI treatment. It has been shown that the addition of a behaviorally silent 5-HT_1A_–receptor antagonist to an SSRI can generate acute inhibitory effects on male rat sexual behavior. Atlas987 is a selective 5-HT_1A_-receptor antagonist with equal potency to displace agonist and antagonist binding to pre- and post-synaptic 5-HT_1A_ receptors in rat and human brain. To investigate whether Atlas987 together with the SSRI paroxetine, a combination called Enduro, induces acute inhibitory effects on male rat sexual behavior, we tested Enduro in Wistar rats in a dose-dependent manner. We first tested the 5-HT_1A_ receptor antagonist Atlas987 in 8-OH-DPAT induced serotonergic behavior in rats. Second, we tested Enduro in a dose-dependent manner in male sexual behavior. Third, we tested the effective time window of Enduro’s action, and lastly, we measured the plasma levels of Atlas987 and paroxetine over an 8-h period. Results showed that Enduro acutely and dose-dependently reduced the number of ejaculations and increased the ejaculation latencies. The behavioral pattern induced reflected a specific effect on sexual behavior excluding non-specific effects like sedation or sensoric-motoric disturbances. The time-window of activity of Enduro showed that this sexual inhibitory activity was at least found in a 1–4 h’ time window after administration. Plasma levels showed that in this time frame both Atlas987 and paroxetine are present. In conclusion, in rats, Enduro is successful in acutely inhibiting sexual behavior. These results may be therapeutically attractive as “on demand” treatment for life-long premature ejaculation in men.

## 1. Introduction

Premature ejaculation is a persistent or recurrent pattern of ejaculation occurring during partnered sexual activity within approximately 1 min following vaginal penetration and before the individual wishes it (Diagnostic and Statistical Manual of Mental Disorders 5th Edition; American Psychiatric Association, 2013). Treatment of premature ejaculation (PE) has been challenging. Apart from psychological approaches, over the last decades many pharmacological treatments have been applied, often with disappointing results. Since the 90-ties, the main developments have been seen with the selective serotonin reuptake blockers (SSRIs), which are prescribed off-label and have to be used chronically (Segraves and Balon, 2014; Waldinger, 2018). Since the seminal studies of Waldinger et al. (1994) and Waldinger et al. (1997, 1998a,b, 2001) several SSRIs (in particular paroxetine, sertraline and citalopram) have been double-blind tested and all delayed ejaculation in PE men after chronic administration (review Waldinger et al., 1998a,b). These findings have been replicated and extended and have settled chronic treatment of SSRIs as a proven and adequate treatment of premature ejaculation (McMahon, 2015). However, chronic use of an SSRI with its inbuilt (serotonergic) side effects has numerous disadvantages and the quest for ‘on-demand’ therapy for PE has always been on the table. Although dapoxetine has been developed as ‘on-demand’ SSRI for PE (Hutchinson et al., 2012; Jern et al., 2015; Yue et al., 2015), several factors seem to play a role in its limited efficacy. Dapoxetine is claimed as rapid onset/short duration of action SSRI in contrast to the classical SSRIs (McMahon, 2015; Du et al., 2019), but the supporting data are not convincing and side effects are high (Waldinger et al., 2006; Jern et al., 2015; Porst and Burri, 2019). This has led to its limited use and rapid stopping after starting treatment (Mondaini et al., 2013; Waldinger, 2014; Park et al., 2017).

Topical agents presently take an important role in ‘on-demand’ treatment of PE (Butcher et al., 2020). The main principal of all these topical agents (creams, sprays) is local anesthesia of the glans penis that leads to delay of ejaculation. Topical agents are well-accepted options and seem particularly effective in acquired PE (Ciocanel et al., 2019; Butcher et al., 2020). However, topical anesthetics are associated with disturbing side effects, including loss of sensitivity (men and women), loss of erection and initiation (men and women: lasting more than 20 min after application; Martyn-St James et al., 2016). It is at least clear that a potential enormous need exists for an efficient and safe medication for on-demand treatment of in particular lifelong PE. The ejaculation distribution theory by Waldinger (2002) postulates that intravaginal ejaculatory latency time in men acts in a biological variation with premature ejaculating men on the very left side of the distribution curve. In rats, a similar distribution curve exists (Pattij et al., 2005). In a 30-min sexual behavior session rats can be divided into sluggish (0–1 ejaculations), normal (2–3 ejaculations) or rapid (4–5 ejaculations) ejaculating rats. Rapid ejaculating rats are also at the left end of the distribution curve and are therefore a nice model for premature ejaculation in men.

In previous male rat studies on sexual behavior incidental findings indicated that combination of an SSRI with a 5-HT_1A_-receptor antagonist inhibited male sexual behavior acutely (Ahlenius and Larsson, 1999; de Jong et al., 2005, 2006; Looney et al., 2005). This pharmacological finding, obtained by using the ‘classical’ 5-HT_1A_ receptor antagonist WAY100,635 (Fletcher et al., 1993), has never been postulated as a promising lead to treat lifelong PE. Instead, in the late 90s and early decade of the 21st century intense research was performed into a faster onset of action of antidepressants, specifically SSRIs (Artigas et al., 2018), as this was considered the next big step in improving the treatment of depressive patients (Kaufman et al., 2016). However, the theory that adding a 5-HT_1A_-receptor antagonist could accelerate the onset of action of SSRIs did not materialize. Several 5-HT_1A_ receptor ligands (mostly partial agonists and selective 5-HT_1A_-receptor antagonists, including amongst others robalzotan; Farde et al., 2000) and DU125530 (van Steen et al., 1998; Olivier et al., 1999) did not accelerate the onset of action of SSRIs in depressed patients (Scorza et al., 2012; Hjorth, 2016). However, we elaborated on the early findings in rats (Ahlenius and Larsson, 1999; de Jong et al., 2005, 2006; Looney et al., 2005) that a combination of an SSRI with a 5-HT_1A_-receptor antagonist could create a new ‘on-demand’ principle for treatment of premature ejaculation in men. The 5-HT_1A_ receptor antagonist Atlas987 (DU125530) has shown equal potency to displace agonist and antagonist binding to pre- and post-synaptic 5-HT_1A_ receptors in rat and human brain (Scorza et al., 2012). Although it has been tested for its antidepressant effects in combination with fluoxetine in humans, it has never been tested as a treatment for premature ejaculation. In the present study, we explored the combination of Atlas987 + paroxetine (called ‘Enduro’) by testing several aspects of the pharmacological effects of the combination and the separate components of Enduro. 5-HT_1A_-receptor agonists can induce very specific serotonergic behaviors like lower lip retraction, flat body posture, forepaw treading and arched back (Berendsen et al., 1989, 1990). In the first experiment we tested whether Atlas987 was able to block 5-HT_1A_-receptor agonist (±)-8-OH-DPAT-induced serotonergic behavior. In the next experiments we explored the effects of Atlas987 on male rat sexual behavior. Acute oral administration of a dose range of Atlas987 combined with a limited range of oral doses of the SSRI paroxetine was tested on male rat sexual behavior of well-trained rats. We hypothesized this combination to acutely inhibit the sexual performance. To assess the best timeframe for inhibiting sexual behavior, we tested a fixed combination of orally administered Atlas987 and paroxetine 1, 2, 3, and 4 h after administration. We expected a time-dependent effect of male rat sexual behavior inhibition. Lastly, to know whether the time of testing reflected detectable plasma levels of both Atlas987 and paroxetine, we measured plasma levels of both drugs up to 8 h after administration in experiment 4.

## 2. Materials and methods

### 2.1. Animals

All rats had *ad libitum* access to food and water and were housed at a 12 h/12 h light dark rhythm (lights on at 7:00 a.m.). Rats were housed 2–4 per cage. Wooden gnawing blocks were provided in the cage for cage enrichment. All animal experimental procedures were approved by the Institutional Animal Care and Use committee of the University of Groningen and were conducted in agreement with the law on Animal Experiments of the Netherlands.

**Figure 1:**
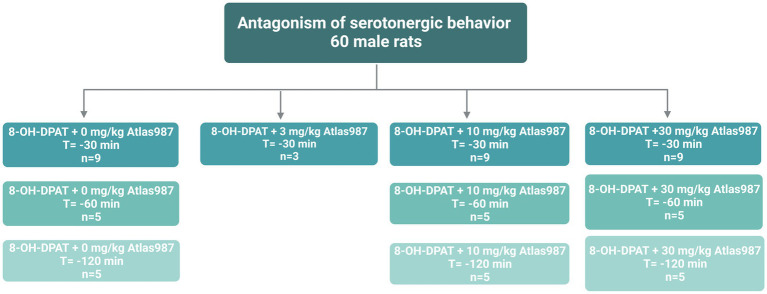
Overview rats used for experiment 1: Antagonism of serotonergic behavior.

For experiment 1: “Antagonism of serotonergic behavior induced by the 5-HT_1A_-receptor agonist (±)-8-OH-DPAT: Dose response and efficacy of oral treatment with Atlas987” 60 male Wistar Unilever rats were used. At arrival rats were approximately 3 months old. Before the experiment started, rats habituated for at least 2 weeks to the animal facility. Male rats were injected s.c. with the 5-HT_1A_-receptor agonist (±)**-**8-OH-DPAT (0.5 mL/kg, 0.5 mg/kg) and the efficacy of the antagonizing effects of Atlas987 on the (±)**-**8-OH-DPAT-induced serotonergic behaviors was tested (Figure 1). The doses of 0, 3, 10 and 30 mg/kg Atlas987 were tested. Rats were orally treated with Atlas987 with flexible PC feeding tubes (Vygon) either 30, 60, or 120 min prior to the (±)-8-OH-DPAT injections. The first group of animals that were injected 30 min before the (±)**-**8-OH-DPAT injection existed of: *n* = 9 for 0 mg/kg Atlas987; *n* = 9 for 10 mg/kg Atlas987 and *n* = 10 for 30 mg/kg Atlas987. Of note: 2 rats died due to wrong oral gavage treatment and 1 rat was wrongly injected with (±)**-**8-OH-DPAT. Also 4 rats were tested for the 3 mg/kg Atlas987, but it became clear that this dose was not efficient enough; therefore, we decided to perform a pilot study on applying Atlas987 60 min before 8-OH-DPAT injections. This resulted in the second group [application of Atlas987 60 min before (±)**-**8-OH-DPAT injections]. This group existed of: *n* = 5 for 0 mg/kg PO Atlas987; *n* = 5 for 10 mg/kg PO Atlas987 and *n* = 5 for 30 mg/kg PO Atlas987. The last group tested was the group that was injected with Atlas987 120 min before the (±)**-**8-OH-DPAT injection. This group existed of: *n* = 5 for 0 mg/kg PO Atlas987 and *n* = 5 for 30 mg/kg PO Atlas987.

**Figure 2:**
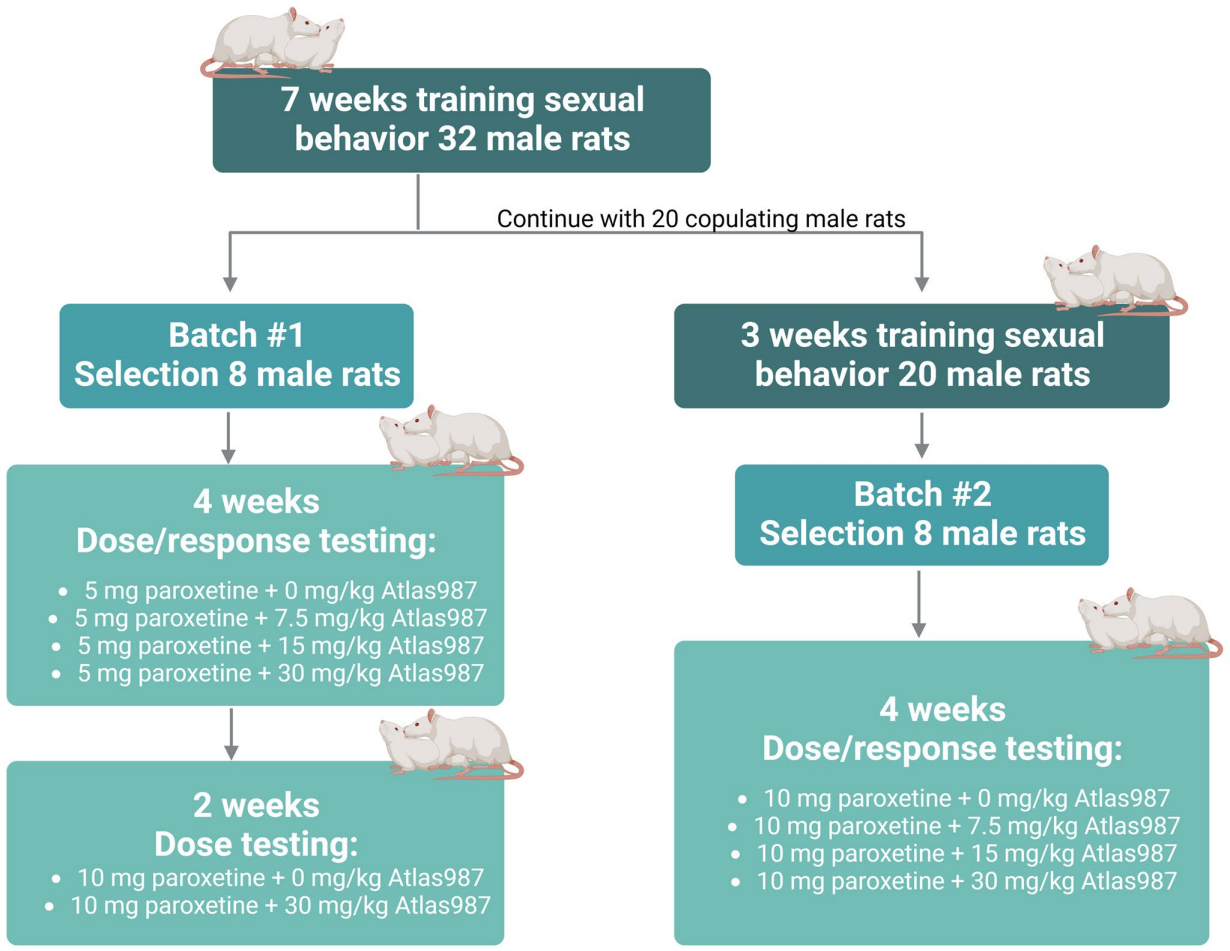
Overview rats used for experiment 2: oral dose–response study of Atlas987 with a fixed oral dose of paroxetine.

For experiment 2: *“oral dose–response study of Atlas987 with a fixed oral dose of paroxetine”* a dose–response study of orally administered Atlas987 combined with fixed oral doses of paroxetine (either 5 or 10 mg/kg) was performed. Adult male Wistar Unilever rats (*n* = 32, 3 months of age) and female Wistar rats (*n* = 40) were used to assess sexual behavior. Because of the Covid-lockdown we only could start the experiments when animals were 9 months old. Two batches were used (see Figure 2). We trained 32 male rats extensively by weekly tests in which each male was tested 30 min against a female in estrus, showing proceptive and receptive sexual behavior (Olivier et al., 2006; Bijlsma et al., 2014; Snoeren et al., 2014; Heijkoop et al., 2018; Huijgens et al., 2021). Rats trained this way gain an individual and stable level of sexual behavior after 4–7 tests (measured by the number of ejaculations/test and the first ejaculation latency; Olivier et al., 2022). We selected male rats with a normal to high level of sexual activity (2–5 ejaculations in the 30-min test) for our pharmacological experiments as described before (Pattij et al., 2005; Chan et al., 2008). Eight rats were used to test 5 mg/kg paroxetine combined with 0, 7.5, 15, and 30 mg/kg Atlas987 in a randomized design (batch #1). Immediately after these treatments, rats of batch #1 were tested another 2 weeks where they received 10 mg/kg paroxetine combined with either 0 or 30 mg/kg Atlas987 in a randomized fashion. The remaining 20 rats (we only continued with rats that were copulating) were trained another 3 times with a two-week interval (total 10 times) because we were unable to test for several weeks. Eight rats were selected to test 10 mg/kg paroxetine combined with 0, 7.5, 15, and 30 mg/kg Atlas987 (batch #2).

**Figure 3:**
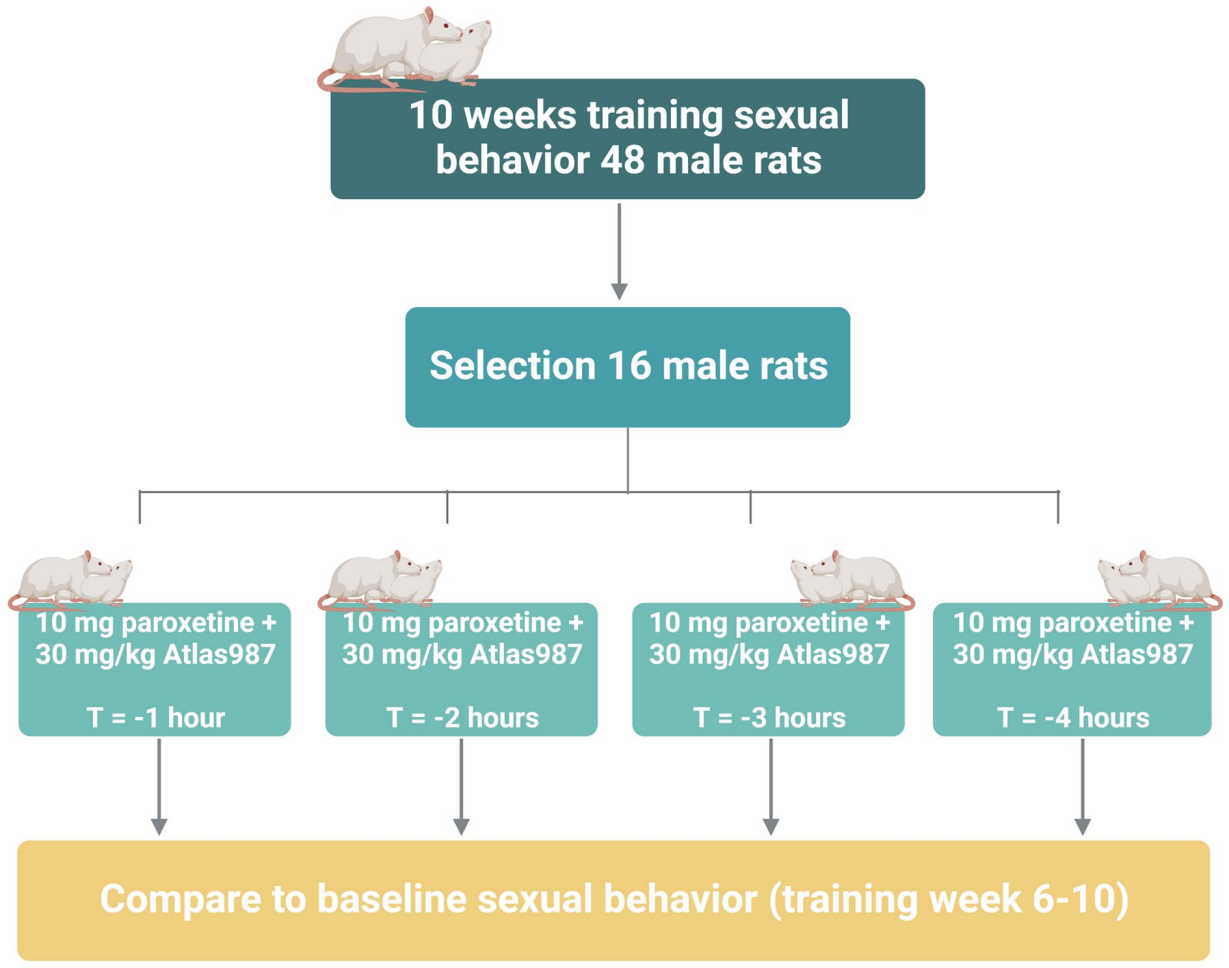
Overview of rats used in experiment 3: fixed dose study of Atlas987 combined with paroxetine; effects on sexual behavior 1, 2, 3, and 4 h after oral administration.

For experiment 3: fixed dose study of Atlas987 combined with paroxetine; “effects on sexual behavior 1, 2, 3, and 4 h after oral administration,” we studied the effects of Atlas987 + paroxetine combination and its constituents on male rat sexual behavior in well-trained and sexually high-performing rats, 1, 2, 3, and 4 h after administration. Adult male Wistar rats (*n* = 48, 3 months of age) and female Wistar rats (*n* = 60) were used in this study to assess male sexual behavior. Methods and material were similar to that of experiment 2. All rats were trained 10 weeks for sexual behavior. Of the 48 trained rats, 16 were selected based on stable sexual behavior (2–4 ejaculations in the last 5 weeks). All 16 rats underwent testing 1, 2, 3, and 4 h after administration of 30 mg/kg PO, Atlas987 combined with 10 mg/kg PO paroxetine (see Figure 3).

For experiment 4: *“Plasma levels of Atlas987 and paroxetine”* we used 12 adult male Wistar Unilever rats (3 months of age). To get insight in the most optimal time window to test the combination of 10 mg/kg paroxetine (6 animals) and 30 mg/kg Atlas987 (6 animals), we studied the plasma levels of these drugs after oral administration. Blood samples were taken at 15, 30, 60, 120, 240, and 480 min after administration.

### 2.2. Drugs

For experiment 1: Antagonism of serotonergic behavior induced by the 5-HT_1A_-receptor agonist (±)-8-OH-DPAT: Dose response and efficacy of oral treatment with Atlas987. Atlas987 (synthesized by Syncom, Groningen, Netherlands) was dissolved in Maisine cc (provided by Gattefossé, Saint-Priest, France), which was also used as a vehicle treatment at the 0 mg/kg Atlas987 dose. 0, 10, and 30 mg/kg Atlas987 were tested. Drugs were dissolved by adding Maisine cc to Atlas987. The combination was warmed-up till 37°C and kept at this temperature for at least 30 min while stirring. The end concentration was never higher than 7.5 mg/ml (maximum concentration still able to dissolve), and was injected in a volume of 4 ml/kg. 0.5 mg/ml (±)-8-OH-DPAT (Tocris, Bristol, UK) was dissolved in saline and subcutaneously administered (0.5 mL/kg). For Experiment 2: “oral dose–response study of Atlas987 with a fixed oral dose of paroxetine” Atlas987 was dissolved as described in experiment 1. Paroxetine (20 mg paroxetine as HCl hemihydrate/ pill; Pharmachemie BV, the Netherlands) was dissolved in sterile tap water. For experiment 3: “fixed dose study of Atlas987 combined with paroxetine; effects on sexual behavior 1, 2, 3, and 4 h after oral administration,” we used Atlas987 and paroxetine as described in experiment 2. In experiment 4 “Plasma levels of Atlas987 and paroxetine” 10 mg/kg paroxetine and 30 mg/kg Atlas987 were injected. Both drugs were used as described in experiment 2.

### 2.3. Tubal ligation in female rats

Female rats had double tubal ligation to prevent pregnancies. For surgery, females were anaesthetized (Isoflurane) and given pain relief subcutaneously (Carprofen, 5 mg/kg) directly before surgery and 24 and 48 h after surgery. Females were at least 3 months old when surgery was performed, and had 2 weeks of recovery before they were made intentionally receptive with estradiol (50 μg in 0.1 mL oil, S.C., 36–48 h before the test) for the sexual behavior tests. Females were used a maximum of once every 2 weeks and no more than 2 times per experimental day.

### 2.4. Behavioral observations

For experiment 1: “Antagonism of serotonergic behavior induced by the 5-HT_1A_-receptor agonist (±)-8-OH-DPAT: Dose response and efficacy of oral treatment with Atlas987” rats were placed in the observation cage immediately after treatment with (±)**-**8-OH-DPAT and their behavior was scored for 60 min. Rats were scored every 5 min for lower lip retraction, flat body posture and arched back. All scores were divided into: 0 = not present; 1 = medium present; 2 = strongly present. All behavior during the 60-min test was video recorded and were also live scored. For Experiment 2: “oral dose–response study of Atlas987 with a fixed oral dose of paroxetine,” sexual behavior training and test sessions were carried out in a red-lighted room between 09:00 AM and 04:00 PM. Male rats were placed in wooden rectangular (57 cm × 82 cm × 39 cm, with a front plexiglas wall) testing boxes filled with a layer of wooden chips bedding material, allowing for non-paced mating. After 10–30 min of habituation, a receptive female rat was introduced into the cage. The receptive female was in the cage for 30 min, while the sexual behavior of the male rat was observed. Each rat received vehicle or a dose of Atlas987, immediately followed by paroxetine (5 or 10 mg/kg) 60 min prior to the sexual behavioral test. The number of mounts, intromissions and ejaculations and the times of the first Mount, Intromission and Ejaculation and the time of the 2nd ejaculation were scored using Boris version-7 software. The mount-, intromission-, and ejaculation latencies and frequencies, the post-ejaculatory interval (PEI) and the intromission ratio (IR: efficiency) [(IR = #I/#M + #I) *100%] were calculated. For experiment 3: “fixed dose study of Atlas987 combined with paroxetine; effects on sexual behavior 1, 2, 3, and 4 h after oral administration,” we trained 48 male Wistar rats for 10 weeks for sexual behavior to stabilize sexual behavior and select male rats that have a stable pattern of 2–5 ejaculations per 30 min, as described in Exp. 2. After these 10 weeks of training, 16 male rats that ejaculated most and also were most stable in their sexual behavior were selected for the experiment (see Figure 3). During the drug experiments 3 rats died. Because the rats displayed a very stable sexual behavior level (in terms of number of ejaculations/test and the 1st ejaculation latency) as control group we decided to use the mean of the five last training tests of these 14 selected rats as comparison to the drug tests, because we otherwise needed 4 extra control groups (corresponds with 4 extra test weeks) which we considered too stressful for the animals. In this experiment all behavior was scored during training tests 1 through 10. We used as baseline the means of all scored of tests 6 till 10 and used them to compare the mean number of mounts and intromissions in the first ejaculation series, the total number of ejaculations and the latency to ejaculate. All behavior during the 30-min test was video-recorded after introduction of the female and were scored using Boris version-7 software. The mount-, intromission-, and ejaculation latencies and frequencies and intromission ratio [=efficiency: IR = #I/(#M + #I) *100%] were calculated.

### 2.5. Plasma collection

Rats were orally administered either 30 mg/kg Atlas987 or 10 mg/kg paroxetine using similar methods as before. After 15, 30, 60, 120, 240, and 480 min a tail cut was used to collect blood samples. Blood was collected using microcuvette (Microcuvette CB 300 μl, K2EDTA, Sarstedt, Netherlands). Blood samples were stored on ice, centrifuged for 10 min at 1500 rcf in a non-cooled centrifuge. Once centrifuged, the samples were stored at −80°C until further analysis with LC–MS/MS.

### 2.6. Bioanalysis of plasma samples

To 20-μl rat K2EDTA plasma, 50-μl of an internal standard work solution was added prior to a protein precipitation procedure using 100 μl acetonitrile. An aliquot of 100-μl of the obtained supernatant was transferred to a 96 deep well plate containing 150-μl of a 0.2% formic acid solution. The deep well plate was vortexed for 1 min after which it was placed in the autosampler at 10°C. The bioanalysis was performed by Ardena Bioanalysis BV, Assen, the Netherlands (Ardena study 21,256). The extracts were injected onto a Shimadzu Nexera HPLC system equipped with a Sciex 6,500+ mass spectrometer from Applied Biosystems. The analytical column used was an Acquity BEH C18 column (1.7 μm, 100 × 2.1 mm). The mobile phase consisted of solvent A: 5% acetonitrile in milli-Q water; solvent B: 1% formic acid, 5% acetonitrile in milli-Q water; solvent C: acetonitrile. The following LC solvent program (0.4 ml/min; 40°C) was used, where solvent B was kept at 10%: [0–0.8 min]: 60% solvent A, 30% solvent C; [0.8–1.6 min]: 35% solvent A, 55% Solvent C; [1.6–3.0 min]: 60% solvent A, 30% solvent C. The tandem mass spectrometry system was operated in positive ion mode. The detailed mass spectrometer conditions were as follows: probe temperature, 650°C; ionization spray voltage, 3,500 V; Turbo Ion Spray gas 1, 30 psi; collisionally activated dissociation gas, nitrogen, adjusted at 10 on a Sciex scale of 0–12; curtain gas, nitrogen, adjusted at 40 psig. The following multiple-reaction monitoring transitions were optimized: m/z 330 → 192.1 (paroxanthine), m/z 336 → 198.2 (paroxanthine-d6, internal standard) and m/z 492 → 196.0 (Atlas987).

### 2.7. Statistical analysis

For experiment 1: “Antagonism of serotonergic behavior induced by the 5-HT_1A_-receptor agonist (±)-8-OH-DPAT: Dose response and efficacy of oral treatment with Atlas987,” a summation of all scores was made for the full hour and a mean ± SEM were calculated. For the first two groups [30 and 60 min Atlas987 application before (±)**-**8-OH-DPAT] a One-way ANOVA was performed. The drug effects of each treatment were compared using Tukey’s multiple comparisons test. For the last group [application of Atlas987 120 min prior to (±)**-**8-OH-DPAT] an unpaired *t*-test was performed. For Experiment 2: “oral dose–response study of Atlas987 with a fixed oral dose of paroxetine,” due to some missing values, mixed-effects model analysis was used. The drug effects of each of the treatment days were compared using Tukey’s multiple comparisons test. For batch #2 a one-way repeated-measures ANOVA was used for the number of ejaculations and for ejaculation latency. Because of missing values, a mixed-effects model analysis was used for the other parameters. Tukey’s multiple comparison test was used for drug effects. For experiment 3: fixed dose study of Atlas987 combined with paroxetine; effects on sexual behavior 1, 2, 3, and 4 h after oral administration,” the sexual behavior parameters were presented as mean ± SEM. Due to some missing values, mixed-effects model analysis was used. The drug effects of each of the treatment days were compared using Tukey’s multiple comparisons test. No statistical analyses were performed on plasma samples in experiment 4 “Plasma levels of Atlas987 and paroxetine.” Graphpad Prism 10.0 was used to perform statistics and for preparing graphs. A *p-*value of 0.05 or smaller was considered significant. Biorender was used for graphical overviews of the experiments.

## 3. Results

### 3.1. Experiment 1: Antagonism of serotonergic behavior induced by the 5-HT_1A_-receptor agonist (±)-8-OH-DPAT: dose response and efficacy of oral treatment with Atlas987

**Figure 4:**
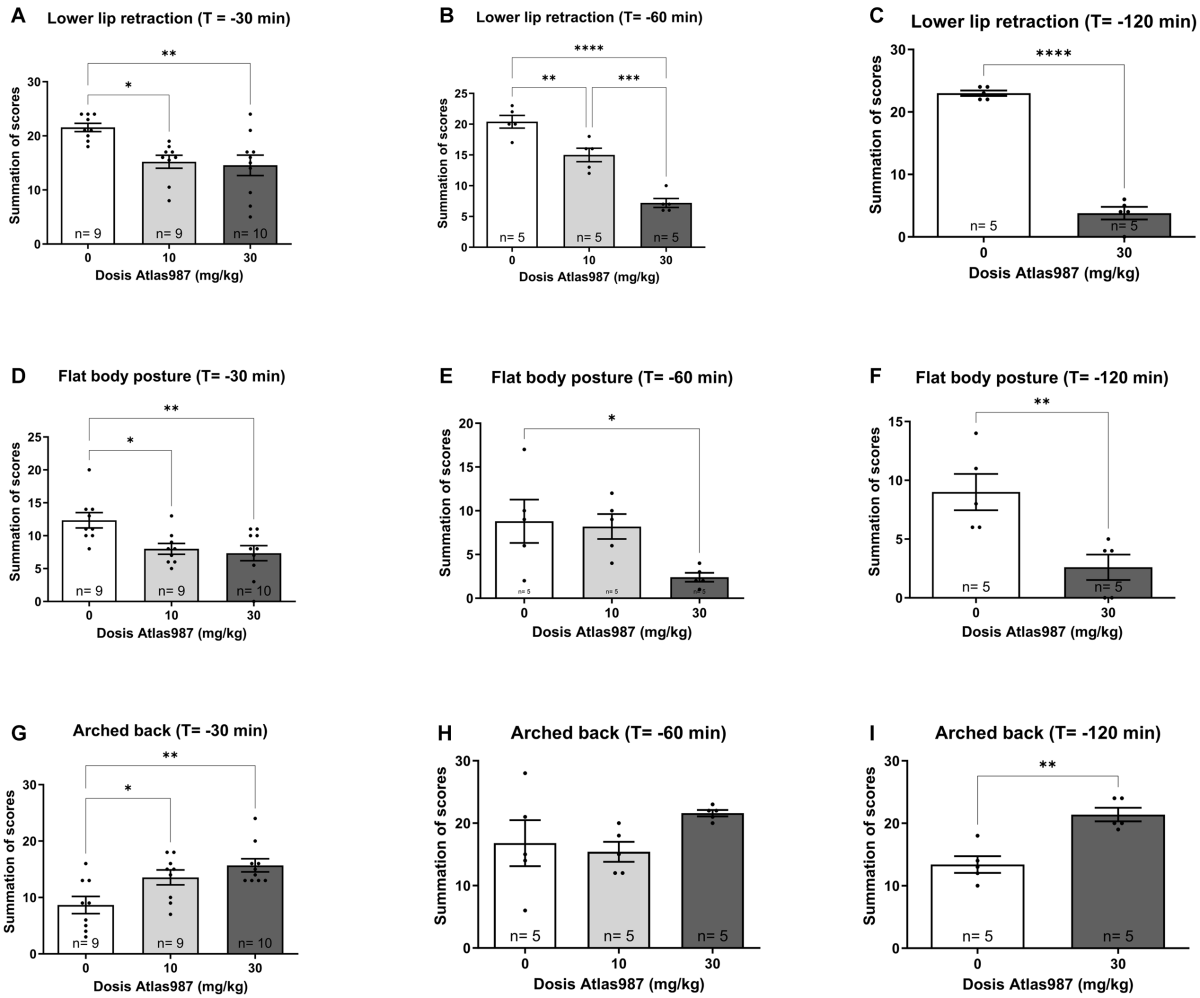
Overview of the antagonizing effects of Atlas987 on (±)-8-OH-DPAT induced lower lip retraction **(A–C)**, Flat body posture **(D–F)** and arched back **(G–I)**. *Significant difference < 0.05; **significant difference < 0.01; ***significant difference < 0.001; ****significant difference < 0.0001.

A significant effect was found on lower lip retraction after treatment with Atlas987 [Figure 4A; *F*(2,25) = 7.391; *p* = 0.0030]. A significant difference between 0 mg/kg Atlas987 and 10 mg/kg Atlas987 (*p* = 0.0123) and between 0 mg/kg Atlas987 and 30 mg/kg Atlas987 (*p* = 0.0045).

Also a significant effect was found on flat body posture after treatment with Atlas987 [Figure 4D; *F*(2,25) = 6.415; *p* = 0.0056]. A significant difference between 0 mg/kg Atlas987 and 10 mg/kg Atlas987 (*p* = 0.0238) and between 0 mg/kg Atlas987 and 30 mg/kg Atlas987 (*p* = 0.0072).

Finally, a significant effect was found on arched back behavior after treatment with Atlas987 [Figure 4G; *F*(2,25) = 7.365; *p* = 0.0031]. 30 mg/kg Atlas987 significantly reduced arched back behavior compared with 10 mg/kg Atlas987 (*p* = 0.0437) and 0 mg/kg Atlas987 (*p* = 0.0025).

#### 3.1.1. Group 2: oral Atlas987 application 60 min prior to subcutaneous (±)-8-OH-DPAT treatment

A significant effect was found on lower lip retraction after treatment withAtlas987 [Figure 4B; *F*(2,12) = 47.19; *p* < 0.0001]. A significant difference between 0 mg/kg Atlas987 and 10 mg/kg Atlas987 (*p* = 0.0050), between 0 mg/kg Atlas987 and 30 mg/kg Atlas987 (*p* < 0.0001) and also between 10 mg/kg Atlas987 and 30 mg/kg Atlas987 (*p* = 0.0003). Also a significant effect was found on flat body posture after treatment with Atlas987 [Figure 4E; *F*(2,12) = 4.44; *p* = 0.0360]. A significant difference was found between 0 mg/kg Atlas987 and 30 mg/kg Atlas987 (*p* = 0.0474). Finally no significant effect was found on arched back behavior after treatment with Atlas987 [Figure 4H; *F*(2,12) = 1.939; *p* = 0.1864].

#### 3.1.2. Group 3: oral Atlas987 application 120 min prior to subcutaneous (±)-8-OH-DPAT treatment

A significant effect was found on lower lip retraction after treatment with Atlas987 [Figure 4C; *T*(1,8) = 17.24; *p* < 0.0001]. 30 mg/kg Atlas987 significantly reduced the lower lip retraction compared with 0 mg/kg Atlas987. Also a significant effect was found on flat body posture after treatment with Atlas987 [Figure 4F; *T*(1,8) = 3.392; *p* = 0.0095]. 30 mg/kg Atlas987 significantly reduced the flat body posture compared with 0 mg/kg Atlas987. Finally a significant effect was found on arched back behavior after treatment with Atlas987 [Figure 4I; *T*(1,12) = 4.682; *p* < 0.0016]. 30 mg/kg Atlas987 significantly lowered the arched back behavior compared with 0 mg/kg Atlas987.

### 3.2. Experiment 2: oral dose–response study of Atlas987 with a fixed oral dose of paroxetine

**Figure 5:**
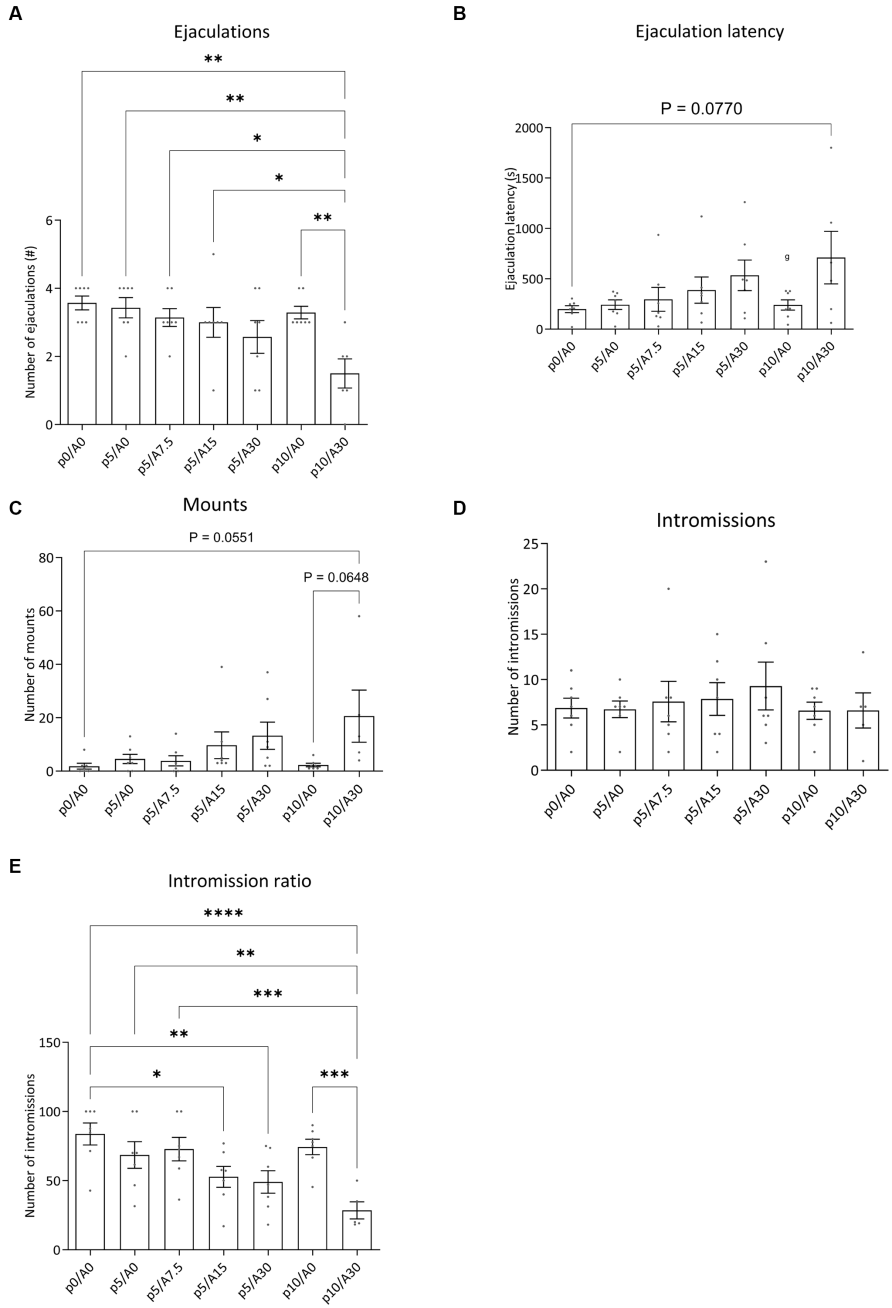
Sexual behavior of male Wistar rats treated with paroxetine (P) and Atlas987 **(A)**. Data are shown as mean ± SEM. The number of ejaculations **(A)**, ejaculation latency time **(B)**, number of mounts to first ejaculation **(C)**, number of intromissions to first ejaculation **(D)** and intromission ratio **(E)** are given. All rats have been randomly treated with vehicle paroxetine+ vehicle Atlas987 (p0A0), paroxetine 5 mg/kg + vehicle Atlas987 (p5/A0), paroxetine 5 mg/kg + 7.5-mg/kg Atlas987 (p5/A7.5), paroxetine 5 mg/kg + 15 mg/kg Atlas987 (p5/A15), paroxetine 5 mg/kg + 30-mg/kg Atlas987 (p5/A30), paroxetine 10 mg/kg + vehicle Atlas987 (p10/A0) and paroxetine 10 mg/kg + 30 mg/kg Atlas987 (p10/A30). All animals received oral treatment 1 h before starting the sex test. *Significant difference < 0.05; **significant difference < 0.01; ***significant difference < 0.001; ****significant difference < 0.0001.

During the course of the experiment of batch #1, we lost two rats after the oral injection. Administration of paroxetine in combination with Atlas987 had significant effects on the sexual behavior of male rats (Figure 5). An overall effect was found for number of ejaculations [*F*_(6, 35)_ = 4.768; *p* = 0.0012], number of mounts [*F*_(6,34)_ = 2.630; *p* = 0.0334], Intromission Ratio [F_(6,34)_ = 8.963; *p* < 0.0001]. A tendency was found for the ejaculation latency [*F*_(6,35)_ = 2.329; *p* = 0.0536], while no significant differences were found for the number of intromissions [*F*_(6, 34)_ = 0.5135; *p* = 0.7938].

#### 3.2.1. Ejaculations

Compared with 0 mg/kg paroxetine combined with 0 mg/kg Atlas987 a significant reduction in ejaculations was found after 10 mg/kg paroxetine combined with 30 mg/kg Atlas987 (*p* = 0.0010). In addition, 10 mg/kg paroxetine combined with 30 mg/kg Atlas987 also significantly reduced the number of ejaculations compared to 10 mg/kg paroxetine combined with 0 mg/kg Atlas987 (*p* = 0.0064). Similar reductions in ejaculations were found when 5 mg paroxetine combined with 0 mg/kg Atlas987 was compared with 10 mg/kg paroxetine combined with 30 mg/kg Atlas987 (*p* = 0.0026), when 5 mg/kg paroxetine combined with 7.5 mg/kg Atlas987, and when 5 mg/kg paroxetine combined with 15 mg/kg Atlas987was compared to 10 mg/kg paroxetine combined with 30 mg/kg Atlas987 (*p* = 0.0150). All other groups were not significantly different from each other.

#### 3.2.2. Ejaculation latency

The 10 mg/kg paroxetine combined with 30 mg/kg Atlas987 showed a tendency to increase the ejaculation latency compared with 0 mg/kg paroxetine combined with 0 mg/kg Atlas987 (*p* = 0.0770). All other groups were not significantly different from each other.

#### 3.2.3. Mounts

A tendency for an increased number of mounts was found after treatment with 10 mg/kg paroxetine combined with 30 mg/kg Atlas987 compared to 0 mg/kg paroxetine combined with 0 mg/kg Atlas987 (*p* = 0.0551) and compared to 10 mg/kg paroxetine combined with 0 mg/kg Atlas987 (*p* = 0.0648). All other groups were not significantly different from each other.

#### 3.2.4. Intromission ratio

The intromission ratio was reduced after treatment with 5 mg/kg paroxetine combined with 15 mg/kg Atlas987 compared to 0 mg/kg paroxetine combined with 0 mg/kg Atlas987 (*p* = 0.0126). Also after treatment with 5 mg/kg paroxetine combined with 30 mg/kg Atlas987 a significant lower intromission ratio was found compared with 0 mg/kg paroxetine combined with 0 mg/kg Atlas987 (*p* = 0.0039). After treatment with 10 mg/kg paroxetine combined with 30 mg/kg Atlas987 a significant lower intromission ratio was found compared with 0 mg/kg paroxetine combined with 0 mg/kg Atlas987 (*p* < 0.0001), compared to 5 mg/kg paroxetine combined with 0 mg/kg Atlas987 (*p* = 0.0013), compared to 10 mg/kg paroxetine combined with 0 mg/kg Atlas987 (*p* = 0.0002), and compared to 5 mg/kg paroxetine combined with 7.5 mg/kg Atlas987 (*p* = 0.0004).

In batch #2, administration of paroxetine in combination with Atlas987 had marginal significant effects on the sexual behavior of male rats (data not shown). An overall effect was found for number of ejaculations [*F*_(4,28)_ = 3.486; *p* = 0.0197] and the ejaculation latency [*F*_(4,28)_ = 3.417; *p* = 0.0214]. No effects were found for the number of mounts [*F*_(4,24)_ = 1.440; *p* = 0.2513], intromissions [F_(4,24)_ = 0.8219; *p* = 0.5240], or intromission ratio [*F*_(4,24)_ = 0.1863; *p* = 0.9432].

*Ejaculations:* 10 mg/kg paroxetine combined with 7.5 mg/kg Atlas987 significantly reduced the number of ejaculations compared with 10 mg/kg paroxetine combined with 0 mg/kg Atlas987 (*p* = 0.0149). A tendency for 10 mg/paroxetine combined with 7.5 mg/kg Atlas987 for lowering the number of ejaculations was also found compared with 0 mg/kg paroxetine combined with 0 mg/kg Atlas987 (*p* = 0.0510).

*Ejaculation latency:* 10 mg/kg paroxetine combined with 7.5 mg/kg Atlas987 significantly increased the ejaculation latency compared with 10 mg/kg paroxetine combined with 0 mg/kg Atlas987 (*p* = 0.0137). A tendency for 10 mg/paroxetine combined with 7.5 mg/kg Atlas987 for lowering the number of ejaculations was also found compared with 0 mg/kg paroxetine combined with 0 mg/kg Atlas987 (*p* = 0.0731).

### 3.3. Experiment 3: fixed dose study of Atlas987 combined with paroxetine; effects on sexual behavior 1, 2, 3, and 4 h after oral administration

**Figure 6:**
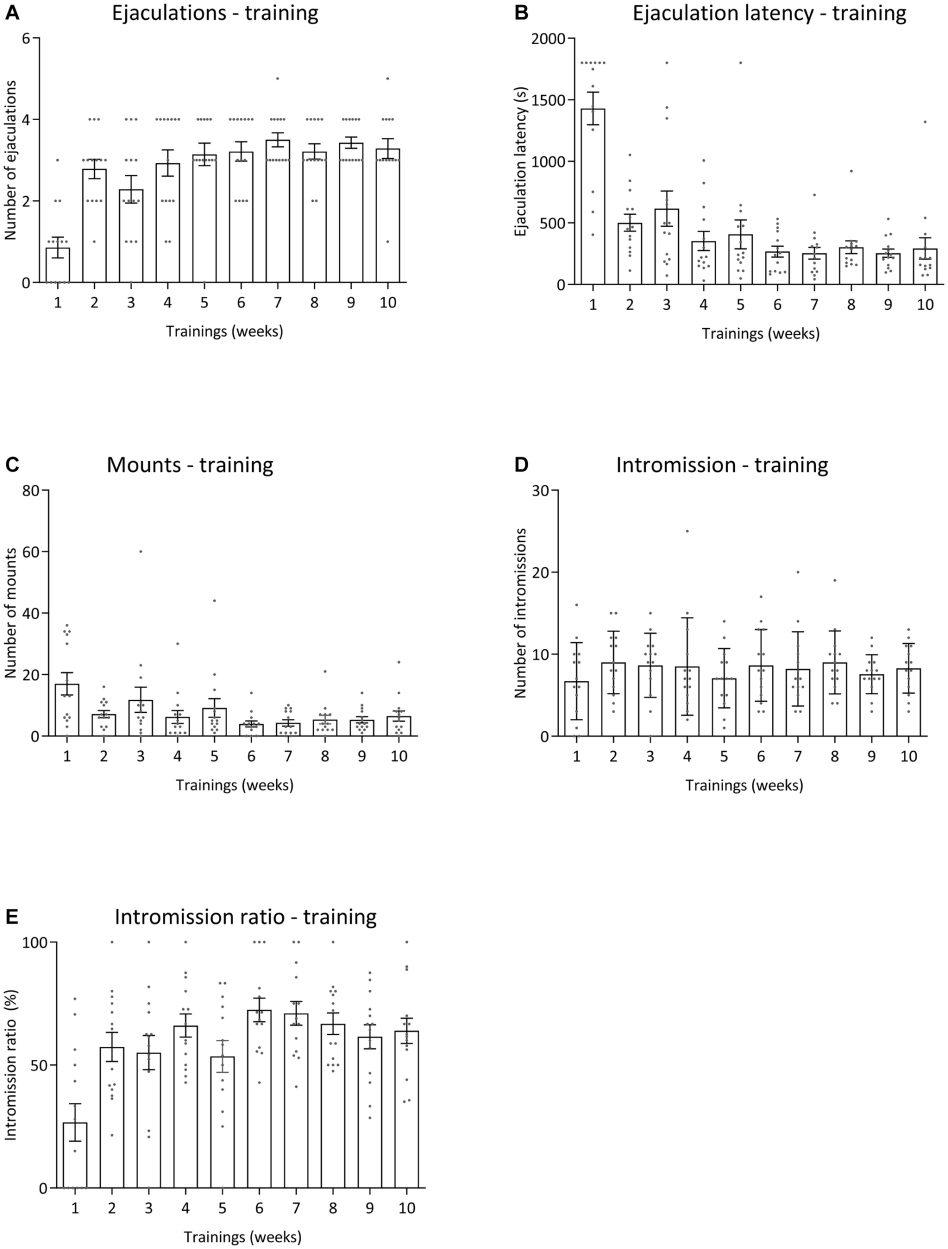
Sexual behavior of male Wistar rats (*n* = 14) during 10 weeks of training. Data are shown as mean ± SEM. The number of ejaculations **(A)**, ejaculation latency time **(B)**, total mounts from start to first ejaculation **(C)**, number of intromissions **(D)**, and intromission ratio **(E)** are given.

**Figure 7:**
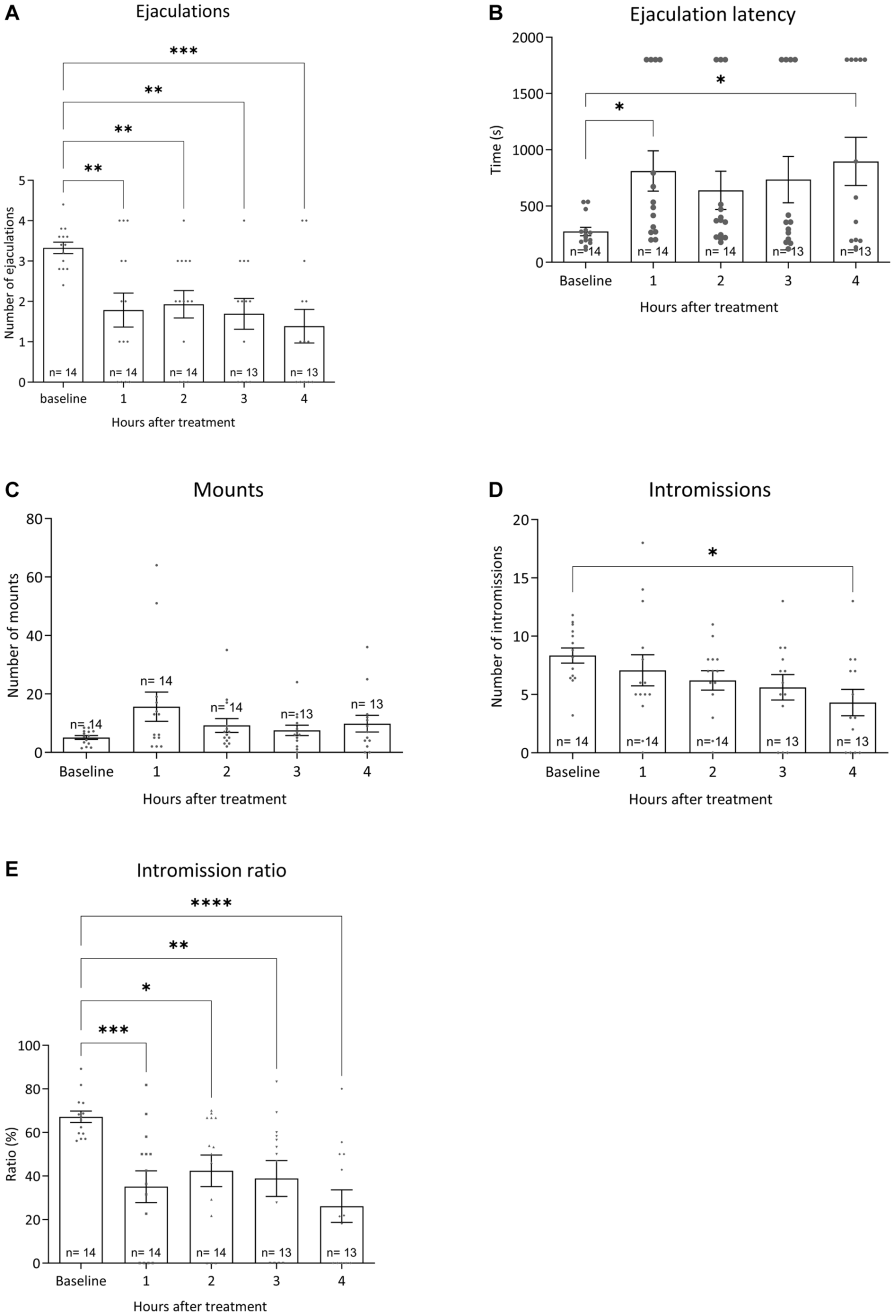
Sexual behavior of Wistar rats in 30-min tests against an estrus female. Baseline data (averages of training weeks 6–10) and the four-time intervals (1, 2, 3, and 4 h) after injecting oral 10 mg/kg paroxetine combined with 30 mg/kg Atlas987 are shown. The number of ejaculations/test **(A)**, the first ejaculation latency **(B)**, the number of mounts before the 1st ejaculation **(C)**, the number of intromissions before the 1st ejaculation **(D)** and the Intromission Ratio **(E)** are shown. *Significant difference < 0.05; **significant difference < 0.01; ***significant difference < 0.001; ****significant difference < 0.0001.

All pharmacological data were compared to baseline sexual behavior during training tests 6 to 10 data for all sexual parameters. Figure 6 displays the results for the various sexual parameters during all the trainings. Unfortunately, we lost 3 rats and data was calculated for 14 animals (and for 1 animal for 3 data points). Figure 7 shows the effects of 10-mg/kg paroxetine (PO) combined with 30-mg/kg Atlas987 (PO) administered 1, 2, 3, or 4 h before testing compared with the baseline [mean data of the training data (6–10 weeks)]. Overall an effect was found for the number of ejaculations [*F*_(4,50)_ = 6.672; *p* = 0.0002], ejaculation latency [F_(4,50)_ = 3.581; *p* = 0.0121], number of intromissions [*F*_(4,50)_ = 3.100; *p* = 0.0235] and intromission ratio [*F*_(4,50)_ = 8.169; *p* < 0.0001]. No overall significances were found for the number of mounts [*F*_(4,63)_ = 1.808; *p* = 0.1384].

*Ejaculations:* Compared with the baseline, treatment with 10-mg/kg paroxetine combined with Atlas987 decreased the number of ejaculations after 1 h (*p* = 0.0035), 2 h (*p* = 0.0097), 3 h (*p* = 0.0026), and 4 h (*p* = 0.0002). *Ejaculation latency:* Compared with the baseline, treatment with 10-mg/kg paroxetine combined with Atlas987 increased the ejaculation latency after 1 h (*p* = 0.0295) and 4 h (*p* = 0.0112). *Number of intromissions:* Compared with the baseline, treatment with 10-mg/kg paroxetine combined with Atlas987 decreased the number of intromissions after 4 h (*p* = 0.0127). *Intromission ratio:* Compared with the baseline, treatment with 10-mg/kg paroxetine combined with Atlas987 decreased the intromission ratio after 1 h (*p* = 0.0006), 2 h (*p* = 0.0127), 3 h (*p* = 0.0055) and 4 h (*p* < 0.0001).

### 3.4. Fourth experiment: plasma levels of Atlas987 and paroxetine

**Figure 8:**
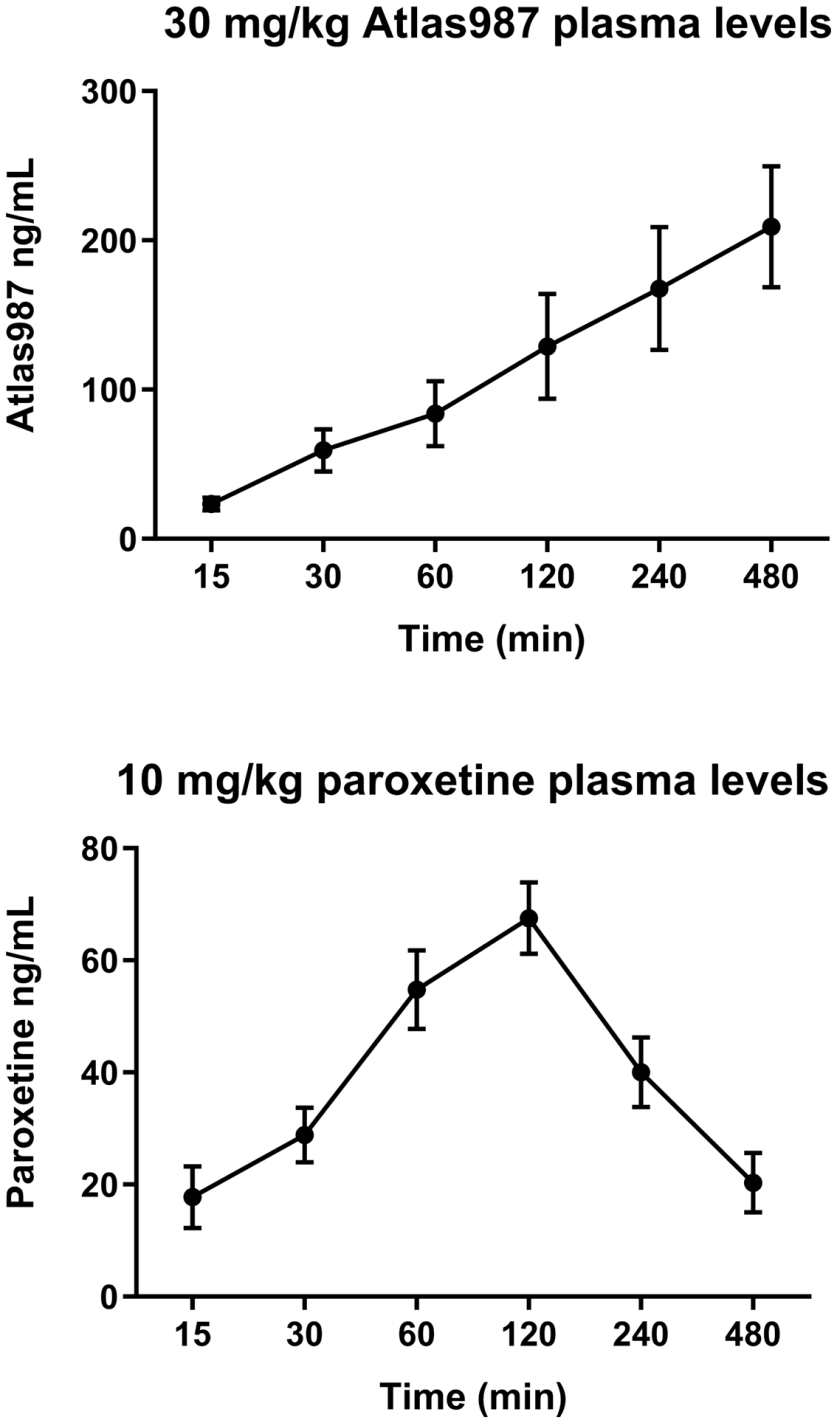
Mean (±SEM) plasma levels (ng/ml) of Atlas987 (top) and paroxetine (bottom) are measured after oral administration of 30-mg/kg Atlas987 and 10-mg/kg paroxetine to male rats (*N* = 6 per group).

Figure 8 shows the plasma levels of orally administered paroxetine and Atlas987. Paroxetine shows an increase in plasma levels up to 120 min after which they decrease quite rapidly (Figure 8, bottom). Atlas987 shows a steady linear increase in plasma levels with a maximum at 8 h. As our behavioral experiments using oral administration were run between 60 and 240 min, plasma levels of Atlas987 and paroxetine were apparently sufficiently high to exert the observed behavioral effects.

## 4. Discussion

We present evidence that combination of the SSRI paroxetine and the 5-HT_1A_-receptor antagonist Atlas987 leads to acute inhibitory effects on male rat sexual behavior. Neither paroxetine alone nor Atlas987 alone when given acutely has inhibitory effects on male rat sexual behavior. We postulate that the paroxetine/Atlas987 combination creates a new approach to on-demand treatment of lifelong Premature Ejaculation. The male rat model used has been extensively used by us (Bijlsma et al., 2014) and has been developed as predictive for rapid ejaculating rats that reflect a rat homolog of a human male with premature ejaculation (Pattij et al., 2005; Olivier et al., 2006, 2022). When testing male rats weekly during 30 min-tests against an estrus female each rat develops his own sexual (endo)phenotype after 5 or more successive tests (Olivier et al., 2017; Esquivel-Franco et al., 2018). In large cohorts (over 3,000 rats tested) male rat’s sexual activity, measured by the number of ejaculations per test, displays an inverted U-shaped distribution curve (Olivier et al., 2022). For testing effects of drugs that inhibit sexual behavior we select rats with high numbers of ejaculation per test. These high performing male rats also display short ejaculation latencies. We used these high performing male rats in the present experiments on sexual behavior.

*Via* oral gavage Atlas987 is able to antagonize the serotonergic syndrome induced by (±)**-**8-OH-DPAT. In general, dose-dependent effects of Atlas987 were found. Overall, the 30 mg/kg has the strongest effects. Moreover, treatment with 30 mg/kg Atlas987 seems to have larger effects when treated 1 or 2 h before (±)**-**8-OH-DPAT injections, indicating that the pharmacological effects of orally administered Atlas987 have to be measured preferably 1–2 h after oral administration. After demonstrating the oral activity of Atlas987 was able to antagonize the serotonergic behavior induced by the 5-HT_1A_-receptor agonist (±)-8-OH-DPAT in experiment 1, we ran a dose–response study in which we studied orally administered Atlas987, orally administered paroxetine and their combination in sexually trained male rats. From previous studies we know that doses of 5 and 10-mg/kg paroxetine have no inhibitory effects on male rat sexual behavior after acute administration (Chan et al., 2008; Bijlsma et al., 2014).

This profile was confirmed in the ensuing study where Atlas987 at orally active doses (as found in the experiments on antagonism of (±)-8-OH-DPAT-induced serotonergic behavior) had no acute inhibitory effects on male rat sexual behavior. Combining inactive doses of paroxetine (5 or 10-mg/kg, oral) with Atlas987 led to dose-dependent acute inhibition of male rat sexual behavior. Combining 10-mg/kg oral paroxetine with higher doses (30-mg/kg oral) of Atlas987 showed rather strong acute inhibitory (on-demand) effects on sexual behavior. At the 5-mg/kg dose of paroxetine, adding up to 30 mg/kg Atlas987 dose-dependently enhanced the sexual inhibition. However, only at the 10-mg/kg dose of paroxetine the adding of 30-mg/kg Atlas987 led to a clear significant sexual inhibitory effect. This inhibition was evident in the enhanced number of mounts till the 1st ejaculation, whereas the number of intromissions till the 1st ejaculation was not affected, leading to a decreased intromission ratio, indicating a decreased efficiency to reach ejaculation. The total number of ejaculations was dose-dependently decreased, whereas the 1st ejaculation latency was dose-dependently enhanced (significant at 10 mg/kg paroxetine combined with 30 mg/kg Atlas987). The total number of mounts over the total 30-min test was dose-dependently enhanced, but no effects were present in the total number of intromissions. It should be noted that a low value of the intromission ratio is related to erectile dysfunction (reviewed in Bialy et al., 2019). We did not measure penile erections, but in the second experiment (Figure 5) we did not see a decrease in the number of intromissions (rather a decrease in the number of mounts). However, in the third experiment we did see a significant decrease in the number of intromissions at the highest dose. Future research should therefore include penile erection observations to confirm that Atlas987 combined with paroxetine is not inducing erectile dysfunctions. Moreover, the highest dose (10 mg/kg paroxetine combined with 30 mg/kg Atlas987) significantly increased the intromission and mount latency (data not shown). This data can be interpreted as evidence that the sexual inhibitory effects of the combination paroxetine/Atlas987 are not caused by general behavioral suppressing effects (e.g., sedation of sensoric/motoric disturbances) as we did not see sedative effects during the tests (observed, not scored). We conclude that specific interfering effects on sexual mechanisms in the brain or spinal cord cause the sexual behavioral inhibitory effects. In a second batch of rats that were trained to further increase the number of animals in the various dose groups no clear behavioral effects were found after comparable pharmacological treatment as in batch #1. Although we do not know the cause, we were confronted with lockdowns due to Covid during this experimental period and were only able to finalize the experiments with relatively old animals that had a very irregular training and treatment schedule. We consider this part of the experiments not valid and will repeat this in the future.

Because we had limited insight in the onset and duration of action of the sexual inhibitory activity of the paroxetine/Atlas987 combination, we performed a study where we injected the effective paroxetine/Atlas987 combination as found in the previous experiments (paroxetine 10-mg/kg PO + Atlas987 30-mg/kg PO) at different injection-test intervals, *viz.* 1, 2, 3, and 4 h before the sexual behavior test. At all times measured, the combination Atlas987/paroxetine exerted a clear sexual inhibitory profile, similar to that observed before, i.e., reduction of the number of ejaculations, enhanced 1st ejaculation latencies, retarded start of first sexual activities (either mount or intromission), enhanced number of mounts without changing or slightly decreasing the number of intromissions which led to decreased efficiency (intromission ratio) of reaching an ejaculation, and lengthened mount and intromission intervals before reaching the 1st ejaculation. This behavioral pattern at all time points measured is in line with the earlier studies after acute administration.

Apparently, simultaneous oral administration of 30-mg/kg Atlas987 with oral 10-mg/kg paroxetine leads to sufficient plasma and brain levels of both molecules to lead to acute inhibitory activity on sexual behavior. We also measured plasma levels of Atlas987 and paroxetine from 15 min till 8 h (Figure 8) after administration, and found at each time point measurable concentrations of each molecule. For paroxetine the highest concentrations were present in the 60–240-min range, with decreasing concentrations at later time points, whereas Atlas987 showed a linear increase over the whole time period.

The present concept of an ‘on-demand’ inhibitor of premature ejaculation, adding a behaviorally silent 5-HT_1A_-receptor antagonist to an acute dose of an SSRI that on itself does not have inhibitory action on male rat sexual behavior illustrates the possibility to develop new approaches in treatment of sexual dysfunctions, in this case lifelong premature ejaculation. Extensive research has shown that SSRIs alone have sexual inhibitory effects in humans and rats but only efficiently after chronic administration (Waldinger et al., 1998a,b; Olivier et al., 1999). Several studies (Chan et al., 2008; Bijlsma et al., 2014) have shown that only chronic, but not acute treatment of SSRIs, leads to inhibition of male rat sexual behavior. Interestingly, 5-HT_1A_-receptor antagonists alone do not influence male rat sexual behavior as shown here with Atlas987 and extensively before with the reference compound WAY100,635 (Ahlenius and Larsson, 1999; de Jong et al., 2005; Looney et al., 2005; Chan et al., 2011; Olivier et al., 2017; Esquivel-Franco et al., 2018). One human study on lifelong PE tested a 5-HT_1A_-receptor antagonist, GSK958108, after an acute single oral dose (Migliorini et al., 2021). The study applied a masturbation model to measure ejaculation latency instead of a stopwatch procedure and the results were not impressive. At a low dose no effects were found and at a higher dose, associated with some side effects, a small, clinically irrelevant increase was found. GlaxoSmithKline discontinued GSK958108 for primary PE (Lacivita et al., 2012). This concurs with our hypothesis that only when a 5-HT_1A_-receptor antagonist is added to an SSRI, an acute clinically relevant inhibitory action on sexual behavior will emerge. It is clear that the acute inhibitory action of ‘Enduro’, as we named the combination of paroxetine and Atlas987, exerts its effects *via* modulation of the serotonergic system.

Because plasma levels of drugs do not necessarily directly relate to neurochemical processes in the brain, insight in the effects of our treatments on 5-HT levels in the CNS would be helpful to possibly better understand the underlying processes in the inhibitory actions of the Atlas987/paroxetine combination on male sexual behavior. SSRIs after acute administration enhance 5-HT levels in various regions of the brain as measured by *in vivo* microdialysis (Fuller, 1994; Beyer and Cremers, 2008). SSRIs have been developed and used as antidepressants and extensive research has investigated the slow onset of antidepressant effects of SSRIs often taking many weeks before clinical efficacy emerges. One of the original theories to advance the onset of antidepressant action of SSRIs was to add an antagonist for the 5-HT_1A_-autoreceptor located on somatodendritic parts of serotonergic neurons (Artigas et al., 2018). Clinically relevant doses of an SSRI increase extracellular levels of 5-HT in the raphe nuclei but less so in the terminal regions in the forebrain. Adding a 5-HT_1A_-receptor antagonist that exerts no intrinsic behavioral effects, to an SSRI strongly enhances 5-HT levels at terminal regions in the forebrain (Sharp and Hjorth, 1990; Gartside et al., 1995; Gardier et al., 1996; Ramaiya et al., 1997; Romero and Artigas, 1997; Sharp et al., 1997; Adell and Artigas, 1998; Hajós-Korcsok et al., 2000; Hjorth et al., 2000; Nakayama, 2002) leading to stimulation of all 5-HT receptors present in these areas. However, 5-HT_1A_-receptor antagonists also blocks postsynaptic 5-HT_1A_-heteroreceptors, abundantly present in many parts of the brain and spinal cord (Sharp and Barnes, 2020; Barnes et al., 2021). We postulate that a certain population of 5-HT_1A_-heteroreceptors, present in brain areas involved in male sexual behavior (e.g., hypothalamus or spinal cord), when blocked, leads to acute inhibition of male rat and human sexual behavior. This mechanism has extensively been investigated in human depression but has not led to a faster onset of action in human depression (Hjorth, 2016; Artigas et al., 2018). It seems plausible that this 5-HT_1A_-receptor blockade is rather specific to male sexual behavior and is not a general (inhibitory) behavioral phenomenon. This is in line with findings that stimulation of 5-HT_1A_-receptors by 5-HT_1A_-receptor agonists has strong and acute pro-sexual effects in rats reflected in shorter ejaculation latencies and higher ejaculation numbers per test (Snoeren et al., 2014; Esquivel-Franco et al., 2020). Moreover, the 5-HT_1A_-receptor agonist 8-OH-DPAT was still able to enhance the sexual behavior of male rats that had reduced sexual behavior after chronic SSRIs (de Jong et al., 2005), whereas in SERT-knockout rats that exhibit an intrinsic low sexual performance (Chan et al., 2011; Esquivel-Franco et al., 2018, 2020), 8-OH-DPAT still exerted pro-sexual effects (Chan et al., 2011; Esquivel-Franco et al., 2018). This strongly suggests that 5-HT_1A_ receptors play an important role in male rat sexual behavior. Since our rat model of premature ejaculation has a strikingly high predictive validity to human male sexual behavior, we postulate that combination of a 5-HT_1A_-receptor antagonist (Atlas987) with an SSRI (paroxetine) creates a new and promising approach in the treatment of on-demand lifelong premature ejaculation in human males. The results show the potential of Enduro as a new approach in the on-demand treatment of lifelong premature ejaculation.

## Data availability statement

The raw data supporting the conclusions of this article will be made available by the authors, without undue reservation.

## Ethics statement

The animal study was approved by the central committee animal experiments in the Netherlands. The study was conducted in accordance with the local legislation and institutional requirements.

## Author contributions

JO, BO, and SP designed the study. JJ and DE-F performed the experiments. JO performed the statistical analysis and drafted the manuscript. BO, SP, JJ, and DE-F critically revised the manuscript. All authors contributed to finalization and approval of the content of the manuscript.

## Funding

This work was supported by the VLAIO (grant No. HBC.2019.2334).

## Conflict of interest

SP and BO were employed by the company Atlas Pharmaceuticals BV.

The remaining authors declare that the research was conducted in the absence of any commercial or financial relationships that could be construed as a potential conflict of interest.

## Publisher’s note

All claims expressed in this article are solely those of the authors and do not necessarily represent those of their affiliated organizations, or those of the publisher, the editors and the reviewers. Any product that may be evaluated in this article, or claim that may be made by its manufacturer, is not guaranteed or endorsed by the publisher.
